# Biphasic mineralized collagen-based composite scaffold for cranial bone regeneration in developing sheep

**DOI:** 10.1093/rb/rbac004

**Published:** 2022-01-18

**Authors:** Jingchuan Zheng, Zhijun Zhao, Yongdong Yang, Shuo Wang, Yonggang Zhao, Yang Xiong, Shuhui Yang, Zhiye Qiu, Tianxi Song, Chunyang Zhang, Xiumei Wang

**Affiliations:** 1 State Key Laboratory of New Ceramics and Fine Processing, Key Laboratory of Advanced Materials of Ministry of Education, School of Materials Science and Engineering, Tsinghua University, Beijing 100084, China; 2 Department of Neurosurgery, The First Affiliated Hospital of Baotou Medical School, Baotou 014010, China; 3 Dongzhimen Hospital Affiliated Beijing University of Chinese Medicine, Beijing 100700, China; 4 Beijing Allgens Medical Science and Technology Co., Ltd., Beijing 100176, China

**Keywords:** mineralized collagen, cranial bone defect, biphasic composite scaffold, developing sheep

## Abstract

Appropriate mechanical support and excellent osteogenic capability are two essential prerequisites of customized implants for regenerating large-sized cranial bone defect. Although porous bone scaffolds have been widely proven to promote bone regeneration, their weak mechanical properties limit the clinical applications in cranioplasty. Herein, we applied two previously developed mineralized collagen-based bone scaffolds (MC), porous MC (*p*MC) and compact MC (*c*MC) to construct a biphasic MC composite bone scaffold (*b*MC) to repair the large-sized cranial bone defect in developing sheep. A supporting frame composed of *c*MC phase in the shape of tic–tac–toe structure was fabricated first and then embedded in *p*MC phase. The two phases had good interfacial bond, attributing to the formation of an interfacial zone. The *in vivo* performance of the *b*MC scaffold was evaluated by using a cranial bone defect model in 1-month-old sheep. The computed tomography imaging, X-ray scanning and histological evaluation showed that the *p*MC phase in the *b*MC scaffold, similar to the *p*MC scaffold, was gradually replaced by the regenerative bone tissues with comprehensively increased bone mineral density and complete connection of bone bridge in the whole region. The *c*MC frame promoted new bone formation beneath the frame without obvious degradation, thus providing appropriate mechanical protection and ensuring the structural integrity of the implant. In general, the sheep with *b*MC implantation exhibited the best status of survival, growth and the repair effect. The biphasic structural design may be a prospective strategy for developing new generation of cranioplasty materials to regenerate cranial bone defect in clinic.

## Graphical Abstract

**Figure:**
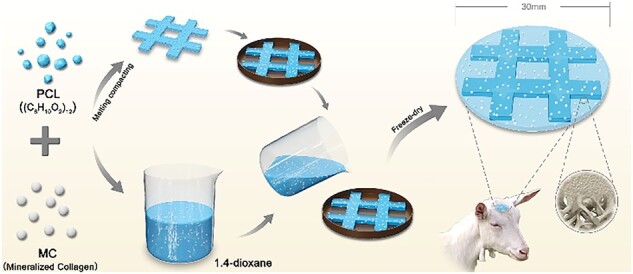

## Introduction

Cranial bone defects that are caused by congenital deformities or acquired injuries, such as trauma, brain and maxillofacial surgery and infection remain a quite common clinical problem [1, 2]. Because of the severe complications resulting from cranial bone defects, such as cranial bone defect syndrome, infection, brain swelling, hydrocephalus, epilepsy, or hemiplegia, and sometimes even lead to psychological and social problems, there Is an extensive consensus that timely and effective repair is critical for patients to provide cerebral protection and reduce risks of accidental injuries, especially for young children with rapidly growing craniums [3–8]. At present, the main strategies of cranioplasty are autologous bone graft, allogeneic bone graft and artificial bone graft substitutes, among which autologous bone graft possesses excellent mechanical properties, osteoconductivity and osseointegration [3–5]. However, autologous bone is generally taken from the patient’s own tibia, ribs, sternum, iliac, which cannot perfectly match the shape of the cranial bone defect and cover the whole defect [6]. Besides, both allograft and xenograft are at the potential risk of immune rejection and transmitting disease, which limits their further applications [7]. Therefore, artificial bone substitutes have gained much attention in cranium repair.

Histologically, natural bone is composed of compact cortical bone and loose cancellous bone. The compact cortical bone mainly provides the mechanical strength of bone, maintains the morphology of bone and resists external impact, while honeycomb-like cancellous bone mainly provides space for cell biological behavior and microenvironment for nutrient transport and metabolite circulation [8–11]. As previously reported, 80% of bone remodeling occurs in cancellous bone [12]. Bone remodeling not only requires porous cancellous bone-like structure, but also relies on suitable mechanical properties of implants that match the surrounding bone [13]. Therefore, it is necessary to develop bone substitutes with excellent osteogenic activity, biodegradability, microporous structure and appropriate biomechanical properties for cranial bone regeneration to achieve timely and effective skull repair, which is essential to improve the life quality of the patients.

A variety of artificial materials have been used to build artificial bone substitutes, such as titanium alloy, bioceramic and polymethylmethacrylate (PMMA) [14]. For example, titanium alloy has good biocompatibility, but it cannot be biodegraded *in vivo* and its mechanical properties are much higher than those of human natural bone, which will lead to cranial bone growth limitation, deformation and atrophy, especially for pediatric patients. And it may produce significant image artifacts in computed tomography (CT) and magnetic resonance imaging, and can damage brain tissue due to its heat conduction [15]. Although PMMA is close to natural bone tissue in mechanical properties, it cannot be biodegraded *in vivo* and has poor binding interface to the defect edge, which limits the regeneration and reconstruction of nascent bone [16]. We previously fabricated biodegradable artificial mineralized collagen (MC) fibrils by biomimetic mineralization method, which imitates not only the composition of natural MC fibrils, but also the hierarchical self-assembly microstructure [17–21]. Based on MC, we previously developed porous MC (*p*MC) bone scaffolds and compact MC (*c*MC) bone scaffolds, which were proved to have good biocompatibility and osteogenic capabilities [22–24].

However, *p*MC scaffold and *c*MC scaffold individually cannot fulfill both the mechanical and osteogenic requirements of cranial bone defect repair during growth and development, so that they cannot treat various complex cranial bone defect cases in clinic [22, 24]. The *c*MC scaffold has no porous structure and its biodegradation is slow, which will hinder the regeneration of nascent bone, while the *p*MC scaffold cannot guarantee sufficient mechanical maintenance during cranial bone regeneration. Therefore, in this study, we constructed a biphasic composite MC scaffold by combining *c*MC scaffold and *p*MC scaffold together with specifically structural design. According to our previous study, *p*MC scaffold could facilitate bone regeneration in the defect center through the dura mater-derived osteogenesis pathway and in the defect edge through the diploic layer osteogenesis pathway, respectively [25, 26]. Therefore, the *c*MC phase was designed as a supporting frame in the shape of tic–tac–toe structure (#) and embedded in *p*MC phase. The biphasic MC (*b*MC) scaffold has interconnected porous structure and suitable mechanical strength, which may provide a synergistic effect on skull regeneration.

Pediatric cranioplasty is still great challenging in clinic because children’s skulls are constantly growing and changing shapes, and with extremely thin cranial bone. It is worth investigating to develop an ideal scaffold for pediatric cranioplasty that must be suitable for cranial bone regeneration and provide enough structural support intraoperation and post-operation [27]. Herein, we developed a large-sized cranial bone defect model using 1-month-old small-tailed Han sheep to simulate children in a fast-growing period. The *b*MC scaffold was used to repair the cranial bone defect in the model, in which the *p*MC phase provided space for the growth of nascent bone and the **#**-shaped *c*MC frame offered the initial mechanical support. The CT imaging and histological examination were performed to investigate the cranial regeneration under the participation of *b*MC scaffolds. The micromechanical properties of nascent bone were evaluated by nano-mechanical testing system.

## Materials and methods

### Preparation of MC scaffolds with different structures

MC powder was prepared as described in our previous work [16], and was used to construct three kinds of scaffolds together with poly(e-caprolactone) (PCL, Jinan Daigang Biomaterial Co., biomedical grade, 300 kDa), including *p*MC/PCL scaffold, *c*MC/PCL scaffold and *b*MC/PCL scaffold.

**Figure 1.**
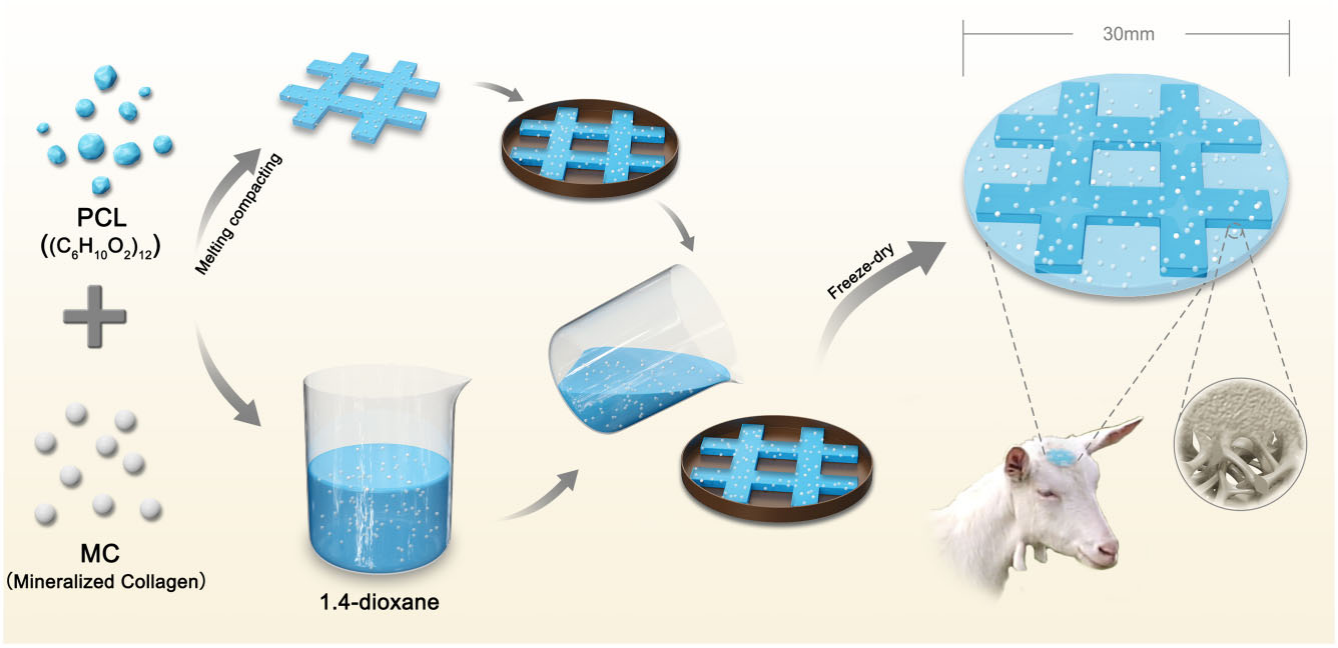
The schematic diagram of preparation and *in vivo* evaluation of *b*MC scaffolds. The *b*MC scaffold was constructed by melting-pressing and freeze-drying. The *c*MC part of the scaffold was constructed by melt pressing PCL and MC, and the porous part of the scaffold was prepared by freeze-drying PCL and MC organic solvents, and the porous part was organically combined with the dense part. After implanted to 30 mm defect of small tail Han sheep, the scaffold promotes the repair of calvarial bone defect and the formation of nascent bone, and ensures the stability of the framework of the long-term defect-repair area.

The *p*MC and *c*MC scaffolds were constructed as previously described [22–24]. The fabrication of the *b*MC scaffold was shown in Fig. 1. For *p*MC, PCL powder (biomedical grade, Jinan Daigang Biomaterial Co., China) was firstly dissolved in 1,4-dioxane with a concentration of 0.1 g/ml. Then, MC powder (0.1 g/ml) was added into the solution, forming a homogenous slurry. Next, the slurry was poured into a designed mold to fabricate the *p*MC scaffold. After being frozen at −20°C, the precursor scaffold was lyophilized to remove the solvent to obtain the *p*MC scaffold. For *c*MC, the PCL powder was melted and mixed homogeneously with MC powder at a ratio of 1:1, then the mixture was shaped in different molds under the pressure of 30 MPa for 10 min, followed by an air-cooling procedure. The *b*MC scaffold was fabricated based on the above procedures. A tic–tac–toe structure of *c*MC scaffold with a frame width of 3 mm was prepared and embedded in the precursor slurry of *p*MC before freeze-drying. After ^60^Co irradiation sterilization, all of the MC/PCL scaffolds were preserved in sterilized condition for further using.

### Measurements on the physical properties of scaffolds

Scanning electron microscopy (SEM) (GEMINISEM, Zeiss, Germany) was used to observe the micro morphology of the prepared scaffolds. In order to maintain the natural state of the internal pore structure, the samples were treated with liquid nitrogen at an ultra-low temperature and then quickly fractured to obtain a fresh section. A platinum layer with a thickness of 5–10 nm was sprayed on the sample surface before observation.

The compressive strength and elastic modulus of the *p*MC, *c*MC and *b*MC scaffolds were measured with a 2000 N load cell using universal mechanical testing machine (SHIMADZU AG-IC, Japan). The samples were prepared as standard cylinders with 10 mm in diameter and 20 mm in length. The load was applied until the three types of scaffolds were compressed to ∼30% of its original length. The compressive modulus was calculated as the slope of the initial linear portion of the stress–strain curve. The compressive strength was determined as the intersection with the line from the 20% strain point with a same slope of elastic modulus. Three individual samples were measured for statistical analysis in each group.

### *In vitro* cytocompatibility of the *c*MC and *p*MC scaffolds

Bone mesenchymal stem cells (BMSCs) (Cyagen Biosciences Inc.) were cultured in glucose Dulbecco’s modified eagle medium with 10% fetal bovine serum and 1% penicillin-streptomycin. The 5 × 10^4^ cells were cultured on different scaffolds in a 48-well plate and incubated in an incubator under an atmosphere of 5% CO_2_ at 37°C. The cell adhesion on the *p*MC and *c*MC scaffolds was examined by SEM (Zeiss, Oberkochen, Germany), as previously described [22–24]. Briefly, the samples were fixed with 2.5% glutaraldehyde in phosphate buffered saline (PBS) after 24-h regular culture, followed by gradient dehydration up to 100% ethanol. Then, the samples were dried through critical point drying (Samdri-PVT-3D, America), and coated with a layer of platinum film for observation by SEM. To evaluate the morphologies of cells, BMSCs were culture on the *p*MC and *c*MC scaffolds for 5 days. Then, the samples were fixed with 4% paraformaldehyde in PBS and stained with rhodamine-phalloidin (1:300; Cat No. PHDR1; Cytoskeleton, Denver, CO, USA) for F-actin as well as DAPI for nuclei. The morphologies were visualized through a laser scanning confocal microscope (Zeiss LSM710, Germany).

### *In vitro* cell culture and osteogenic differentiation induced by MC

To evaluate the osteogenic activity of MC on stem cells, three groups of samples were prepared, including *p*MC scaffold, pure PCL scaffold and hydroxyapatite (HA)/PCL scaffold as control. The HA/PCL scaffold was fabricated by mixing nano-HA and PCL at a ratio of 1:1. SD rat BMSCs (Cyagen Biosciences Inc.) were cultured in complete medium (RASMX-90011, Cyagen Biosciences Inc.) under an atmosphere of 5% CO_2_ at 37°C. Reverse transcription polymerase chain reaction (RT-PCR) was used to evaluate the osteogenesis-related gene expression quantitatively. The concentration of BMSCs seeded on each kind of scaffold was 1.5 × 10^5^ per well in 6-well culture plate. When the cells reached a confluence at about 70%, the complete medium was changed to osteogenic induction medium (RASMX-90021, Cyagen Biosciences Inc.) and the inducing process continued for 2 weeks. The total cellular messenger ribonucleic acid was then isolated and purified via miRcute miRNA Isolation Kit (DP501, TIANGEN BIOTECH CO., LTD), and the complementary deoxyribonucleic acid was obtained using FastQuant RT Kit (KR106, TIANGEN BIOTECH CO., LTD). RT-PCR was performed using iTaq Universal SYBR Green Supermix (172-5121, BIO-RAD) via Thermal Cycler (T100, BIO-RAD) and the relative level of gene expressions including alkaline phosphatase (ALP), Runt-related transcription factor 2 (Runx2), bone morphogenetic protein-2 (BMP-2), osteopontin (OPN), osteocalcin (OCN) and collagen I (Col 1) of BMSCs were measured by Real-Time System (CFX96, BIO-RAD). The data were recorded and then calculated using the 2^−ΔΔCt^ method. The primer sequences (Beijing Genomics Institute, BGI, China) were designed by referring to some similar works related to BMSCs osteogenic differentiation [28–30]. The primer sequences were shown in Supplementary Table S1.

### *In vivo* evaluation of different scaffolds in the regeneration of sheep cranial bone defect model

All of the experimental procedures involving animals were performed in accordance with Guides for the Care and Use of Laboratory Animals from the Chinese Ministry of Public Health and US National Institutes of Health following the IACUC guidelines. The surgeries were carried out at the First Affiliated Hospital of Baotou Medical College of China.

A large-sized cranial bone defect model with a diameter of 30 mm was used to evaluate the performance of the MC scaffolds in developing sheep. Totally 24 healthy 1-month-old sheep were randomly divided into four groups: defects without implantation (blank group), *p*MC scaffold implantation (*p*MC group), *c*MC scaffold implantation (*c*MC group) and *b*MC implantation (*b*MC group). After injection of 3% sodium pentobarbital (30 mg/kg weight), the heads of sheep were shaved and the incisions were made upon the center of calvarias, followed by removing periosteum partially to make the bones exposed. A 30-mm diameter round defect was then drilled by bone drill and the bonesnaps were taken out via byrongeur forceps carefully to ensure the dura mater intact. After the implantation of *p*MC, *c*MC and *b*MC, respectively, the wound was sutured. Penicillin (1 600 000 IU) was injected intramuscularly every day for 5 days postoperatively. The tissue samples including both defect area and surrounding bone were harvest to evaluate the bone regeneration at 1, 3 and 6 months postoperatively.

### CT imaging of defect areas

The whole head of each sheep was observed via CT (Philips, Netherlands) scanning to confirm the situation of defect regeneration as well as the cranial bone developing at immediately post-surgery, 1, 3 and 6 months postoperatively. The data were transformed into three-dimensional (3D) reconstruction images to compare the effects of different implants and the repair outcomes. X-ray coronal scan images were also taken at the same time points as an assistant evaluation of osteogenesis according to the difference of density on images.

### Histological staining and assessments of regenerated bone

The cranial bone samples harvested at 1, 3 and 6 months after surgery were fixed with 4% formaldehyde for 24 h and decalcified with 10% ethylenediaminetetraacetic acid for 6 months. After gradient dehydration, the samples were embedded in paraffin and cut into 4-μm-thickness sections using microtomes (Leica RM2234, Germany). Both the Masson’s trichrome staining and hematoxylin-eosin (H&E) staining were conducted for histological evaluation, and the sections were observed using a light microscope (OLYMPUS IX81, Japan).

### Micromechanical properties of the cranial bone defect area

The surface of harvested samples at 3 and 6 months postoperatively was modified using dental drill and polished to meet the test requirements. A high-precision nano-mechanical testing system (TI9980, Brook, USA) was used to measure the mechanical properties of the neo-bone tissue. In order to achieve statistical results, the number of points selected for each area is over 10.

### Statistical analysis

All results are presented as the mean* *±* *standard deviation (SD). For *in vitro* studies, each experiment was conducted independently at least three times. The normality test was performed using the Kolmogorov–Smirnov test in SPSS (v.23.0; IBM Corp., Armonk, NY, USA). Statistical analysis of normally distributed data was carried out using independent *t*-tests or one-way analysis of variance. Statistical analysis of the data without normal distribution was carried out using a non-parametric method in conjunction with an appropriate *post-hoc* test (least significant difference). Differences were considered statistically significant when *P *<* *0.05, shown as *; *P *<* *0.01, shown as **.

## Results

### Scaffold fabrication and properties

**Figure 2.**
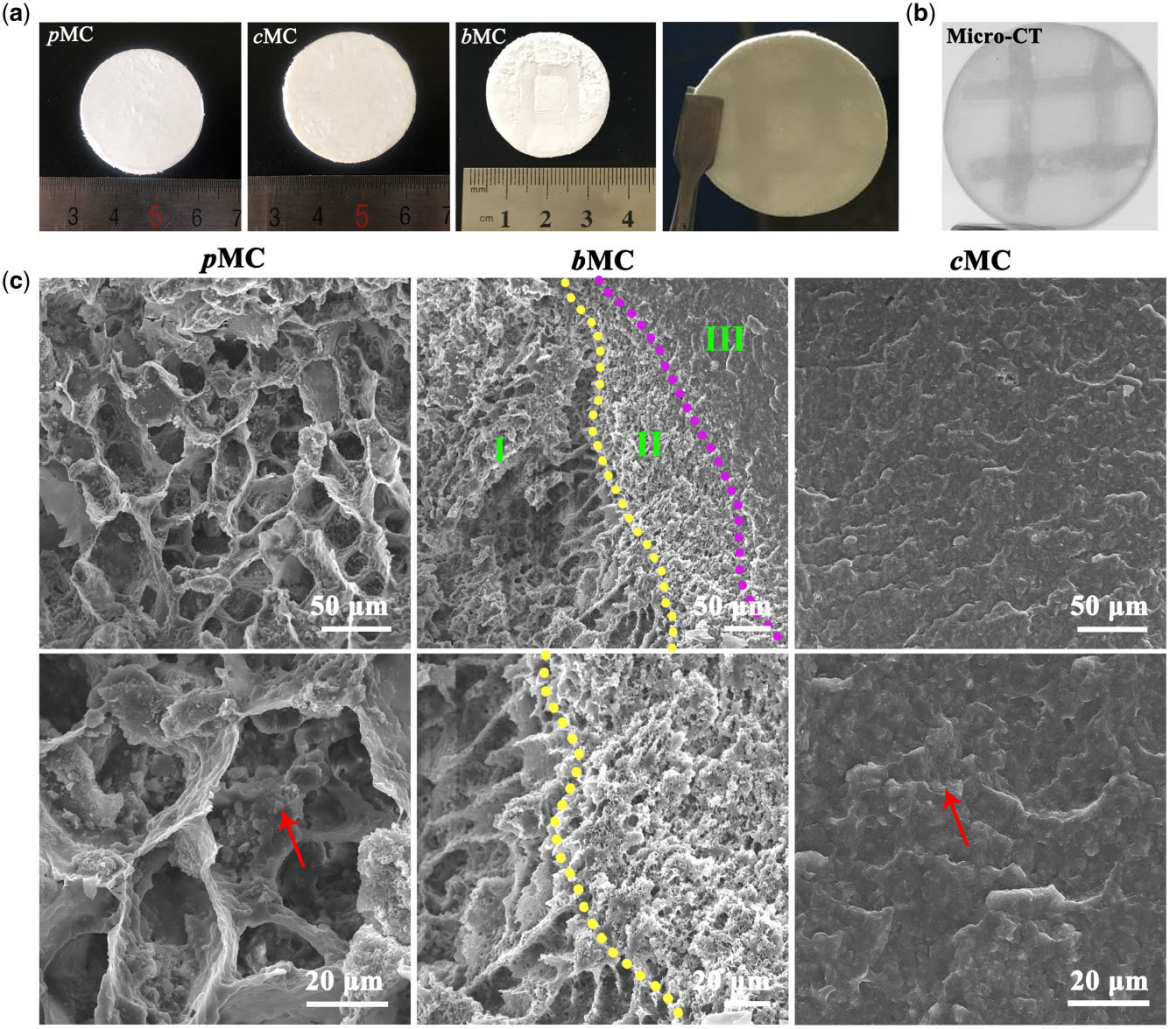
Appearance and microstructure of *b*MC, *c*MC and *p*MC scaffolds (**a**) General appearance of *p*MC, *c*MC and *b*MC scaffolds. (**b**) Micro-CT images of *b*MC scaffold. (**c**) SEM images of cross-sections of *p*MC, *c*MC and *b*MC scaffolds. Arrows refer to the uniformly dispersed MC deposition in *p*MC scaffolds and *c*MC scaffolds, respectively. There are three parts in the *b*MC scaffold: (I) *p*MC phase, (II) transition zone between *p*MC and *c*MC phases and (III) *c*MC phase.

All the scaffolds were shaped into round disks with diameters of 30 mm, as shown in Fig. 2a. The micro-CT image of *b*MC (Fig. 2b) showed different contrasts, with the darker area indicating *c*MC phase and the lighter area indicating *p*MC phase because of the porous structure of the *p*MC phase with low density and the dense structure of the *c*MC phase with high density. The microstructures of *p*MC, *c*MC and *b*MC scaffolds were observed by SEM, respectively (Fig. 2c). The *p*MC scaffold exhibited typical interconnected and hierarchical pore structure, while the *c*MC scaffold showed a compact morphology. In *b*MC scaffolds, there was no sharp boundary between the *p*MC phase (marked as I in Fig. 2c) and *c*MC phase (marked as III in Fig. 2c), but an interfacial bond zone (marked as II in Fig. 2c) instead, where the pore size gradually decreased away from *p*MC phase until disappeared in *c*MC phase. The interfacial bond zone that highly mimicked the interface structure between natural cancellous bone and dense bone contributed to the tight connection between *p*MC and *c*MC phases and the integrity of the *b*MC scaffold. The MC formed clusters with a scale of several microns dispersedly attached on the PCL framework, as marked by red arrows in the high-magnification images.

The mechanical properties of three kinds of scaffolds were determined by the compressive stress–strain curves (Supplementary Fig. S1). As shown in Table 1, the compressive strength and elastic modulus of *c*MC (*σ* = 29.56 ± 1.23 MPa, *E* = 3.17 ± 0.39 GPa) were much higher than those of *p*MC (*σ* = 0.86 ± 0.01 MPa, *E* = 0.05 ±0.01 GPa,), due to the compact microstructure of *c*MC. And the value of compressive strength and elastic modulus of *b*MC is between *p*MC and *c*MC (*σ* = 18.35 ± 0.64 MPa, *E* = 1.13 ± 0.03 GPa). Because the *p*MC and *b*MC scaffolds possessed same compositions, the distinct mechanical properties between them were dominantly attributed to the remarkable differences on the *c*MC phase in the *b*MC scaffold.

**Table 1.** Mechanical properties of natural bone and the *p*MC, *c*MC and *b*MC scaffolds used in this study

	*p*MC	*c*MC	*b*MC	Cancellous bone	Compact bone
Compressive strength (MPa)	0.86 ± 0.01	29.56 ± 1.23	18.35 ± 0.64	1 − 10	100 − 200
Elasticity modulus (GPa)	0.05 ± 0.01	3.17 ± 0.39	1.13 ± 0.03	0.1 − 3	10 − 20

### *In vitro* cytocompatibility of *c*MC and *p*MC scaffolds

**Figure 3.**
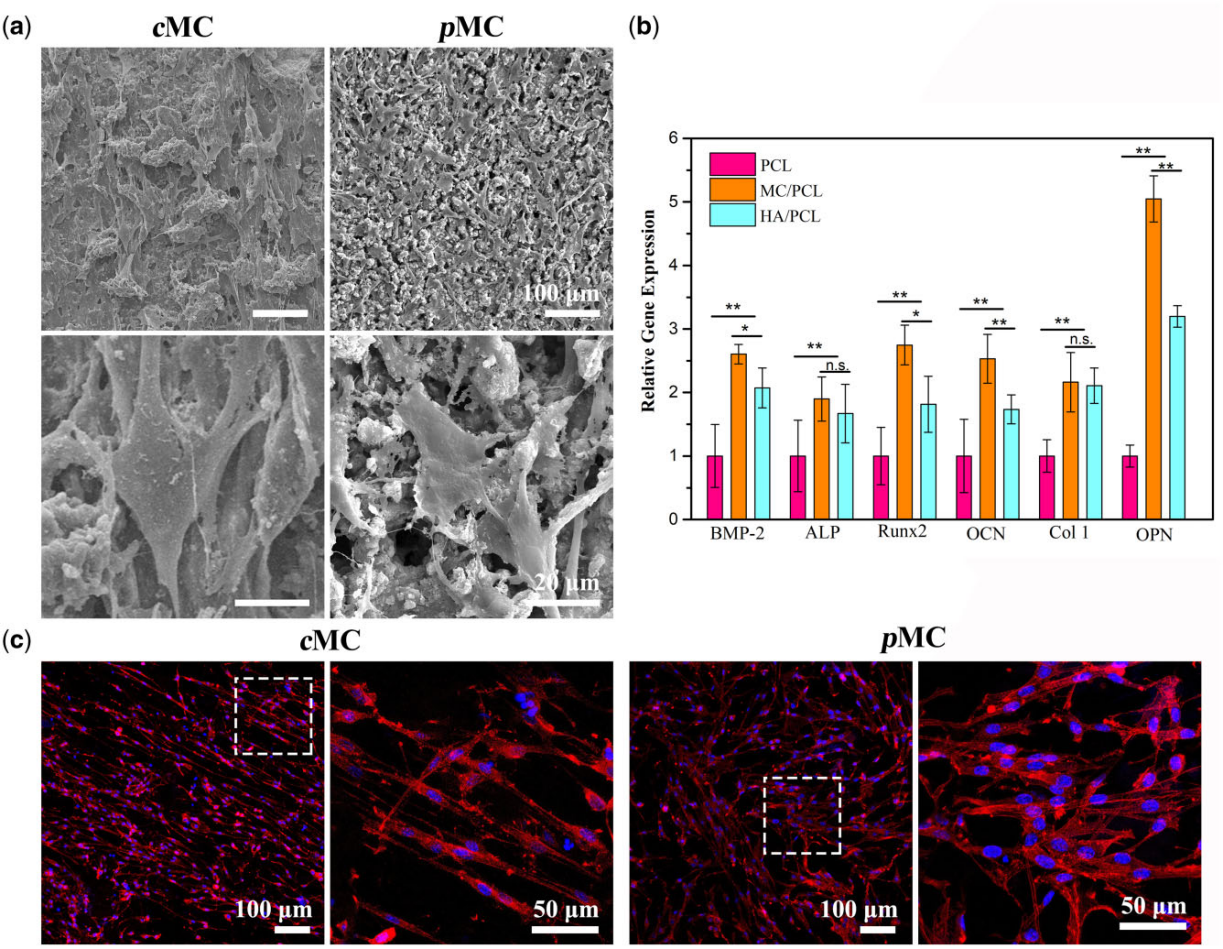
Morphology and osteogenic differentiation of BMSCs on different scaffolds (**a**) Cell morphology on the surface of *c*MC and *p*MC. (**b**) Representative fluorescence microscopy images of BMSCs on *c*MC and *p*MC. (**c**) Gene expression of differentiated BMSCs on PCL, MC/PCL and HA/PCL. Data are presented as mean±SD. *P <* *0.05 and *P <* *0.01.

After 24-h culture, most of the BMSCs attached on the *c*MC and *p*MC scaffolds and maintained viability without dead cells floating in the culture medium. The SEM photographs of morphologies of BMSCs cultured on *p*MC scaffolds indicated that the cells underwent adhesion and spreading, displaying typical spindle cell shape and protruded pseudopods (Fig. 3a). However, the cells on the surface of *c*MC scaffolds showed worse adhesion compared with those on *p*MC, indicated by the more slender cell and the lack of obvious protruded pseudopods. The fibrous pseudopods of cells in *p*MC group were much longer than those in the *c*MC group. Furthermore, as shown in Fig. 3b, the cytoskeletons were revealed by LSM images. BMSCs on both of *c*MC and *p*MC underwent obvious proliferation after 5 days of incubation. The cells on *c*MC scaffolds were poorly spread and the intracellular fibers were entangled. Compared with cells on the *c*MC scaffold, the cells on the *p*MC scaffold spread better, with larger cell body and clearer skeleton fibers in the cytoplasm.

### Osteogenic differentiation of BMSCs induced by MC

To evaluate the osteogenic activity of MC and relevant mechanism, BMSCs osteogenic differentiation on the PCL, HA/PCL and MC/PCL scaffolds after 14 days of culture were evaluated. Pure PCL scaffold and HA/PCL scaffold with HA powders added instead of MC were used as controls. The results showed that the osteogenic-related genes including BMP-2, ALP, Runx2, OCN, Col1 and OPN were significantly up-regulated in the MC/PCL and HA/PCL groups compared with pure PCL group (Fig. 3c). The expression of BMP-2 and Runx2 in the MC/PCL group was significantly higher than that in the HA/PCL group (*P *<* *0.05). Additionally, the MC/PCL group also has obvious advantages over the HA/PCL group in the expression of OCN and OPN genes (*P *<* *0.01).

### CT imaging and evaluation of regenerated bone

**Figure 4.**
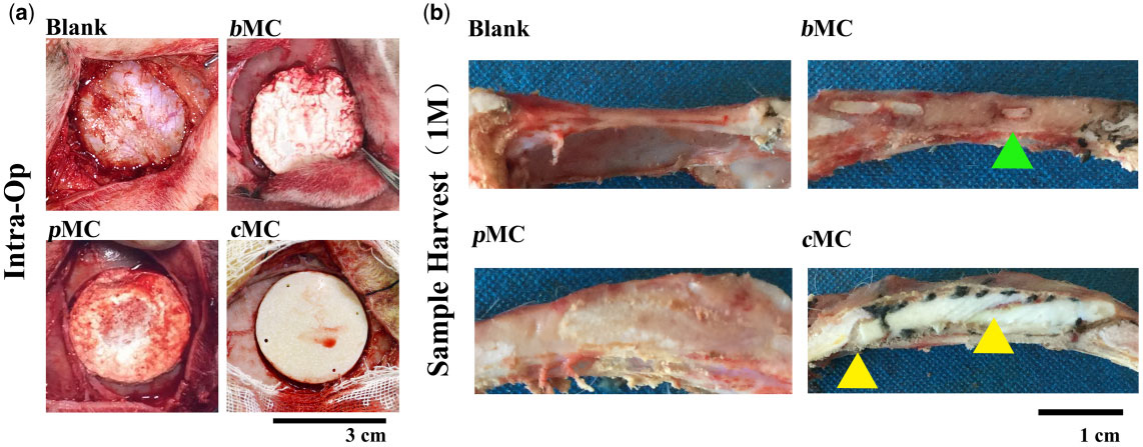
Gross observation intra-operatively and postoperatively (**a**) Construction of 1-month-old sheep cranial bone defect model and scaffold implantation. (**b**) Gross observation of samples harvested at 1 month postoperatively.

The *c*MC, *p*MC and *b*MC scaffolds were implanted to repair 30-mm diameter cranial bone defects (Fig. 4a) and harvested at 1, 3 and 6 months. In order to clearly evaluate the tissue features inner and around the implants, the gross observation on the cross-sectional morphologies at 1 month after the operation was checked and shown in Supplementary Fig. S2 and Fig. 4b. In the blank group, only a thin layer of soft connective tissue existed in the defect area, connecting the original cranial bones at both ends, and there was no obvious sign of bone regeneration. In the *b*MC group, the thickness of the defect area was consistent with that of the peripheral cranial bone, and the original morphology of the scaffold was not observed, and it was difficult to distinguish the contour of the defect area. Touching using a tweezer can feel that the regenerated tissue has a certain hardness. Within the regenerated bone tissue, the existence of the internal *c*MC phase can be observed, and the structure was stable, without obvious degradation but had good integration with the surrounding bone tissues, marked by green arrow. There were also a large number of nascent tissues in the defect area of the *p*MC group, and no material part was observed by the naked eye. However, when using a tweezer, it feels that the hardness is uneven, some parts were soft that may be part of the remaining materials, and some parts were hard similar to bone hardness. In the *c*MC group, it can be observed that the material still existed (yellow arrows) and had good osteointegration with surrounding bone tissues.

**Figure 5.**
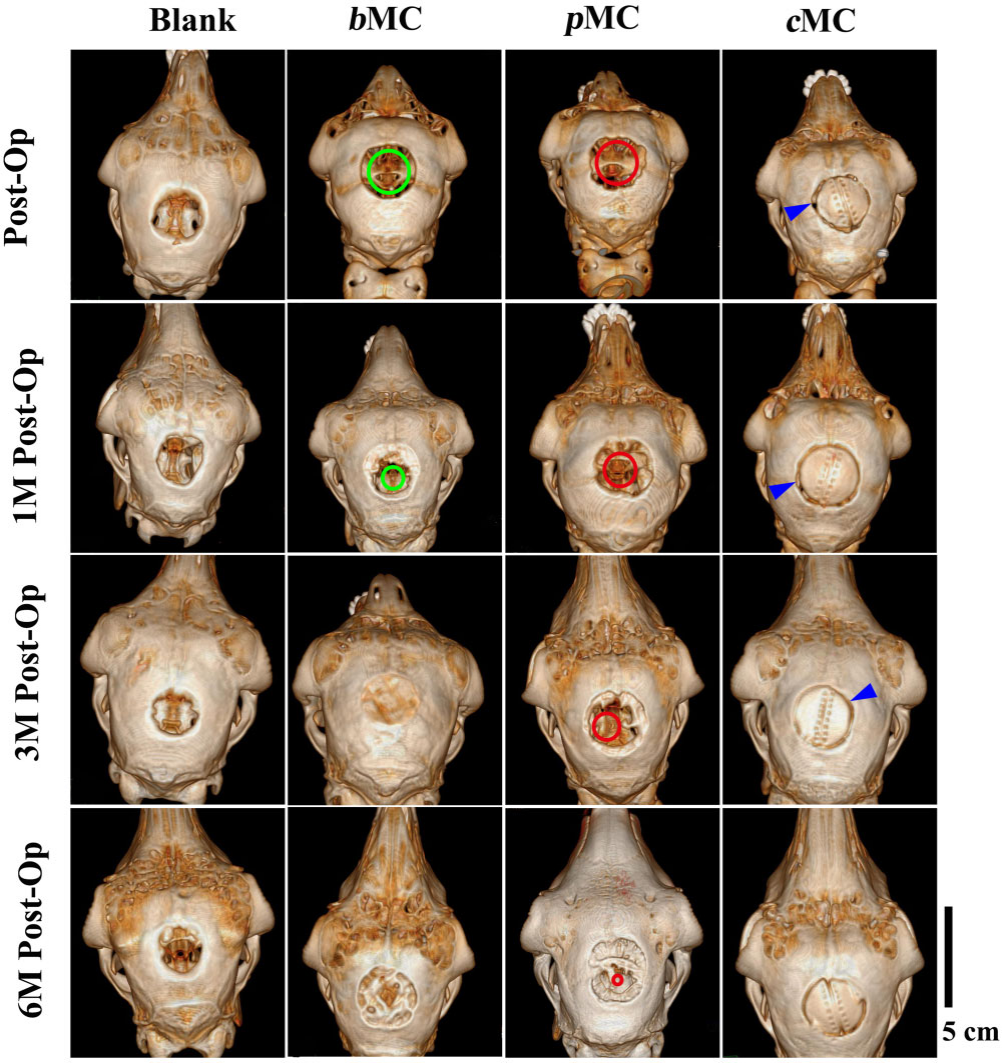
The 3D reconstructed CT images of the defect area in different groups (blank, *b*MC, *p*MC and *c*MC) postoperatively and at 1 month (1M Post-Op), 3 months (3M Post-Op) and 6 months (6M Post-Op) Scale bar = cm. Green circles, red circles and blue arrows represent defect edge position of cranial bone defect in *b*MC, *p*MC and *c*MC groups, respectively.

As shown in Fig. 5, the 3D reconstructed images of 30-mm defect areas in the *b*MC, *p*MC and *c*MC groups at 1, 3 and 6 months after surgery were indicated by the red circles, green circles and the defect edges of *c*MC were signed with blue arrows, respectively. The shape of the *c*MC scaffold had hardly changed and remained high contrast and still covered the entire defect area until 6 months. There was no obvious regeneration of nascent bone tissue in the blank group after 1 month. In the *p*MC group, only a small amount of nascent bone regeneration appeared at the edge of the defect, and in the *c*MC group, bone fusion occurred between more than half of the peripheral bone and the scaffold. While the bone regeneration in the *b*MC group was obvious, forming nascent bone bridge connections, some of which has spanned the whole defect area transversely. Nearly one-third of the defect area in the *b*MC group was covered with nascent bone tissue. At 3 months postoperatively, a small amount of bone regeneration appeared at the edge of the blank group. In the *b*MC group, the entire defect area was fully filled with regenerated bone tissue. However, the shape of the defect area was still similar to that of the implant scaffold and lacked the contour of the natural cranial bone, indicating that the bone tissue formed within the scaffold had no structural remodeling. And the bone regeneration in the *p*MC group was similar to that of the *b*MC group at 3 months after operation, with a certain amount of nascent bone formed. The *c*MC scaffold had basically completed the bone fusion with the peripheral cranial bone, and the boundary between the scaffold and the surrounding bone tissue had partially become blurred. At 6 months, the size of the defect in the blank group had no obvious change compared with that at 3 months, without further nascent bone regeneration. The defect area of the *b*MC group remained covered with nascent bone tissue, however, with more complex structure and the formation of cranial bone contour. In the *p*MC group, the regenerated bone had occupied the vast majority of the total defect area, and basically achieved the closure of the skull. On the basis of bone fusion, there are two additional bone links with the surrounding cranial bone in the *c*MC group, which was a signal for further bone tissue growth.

**Figure 6.**
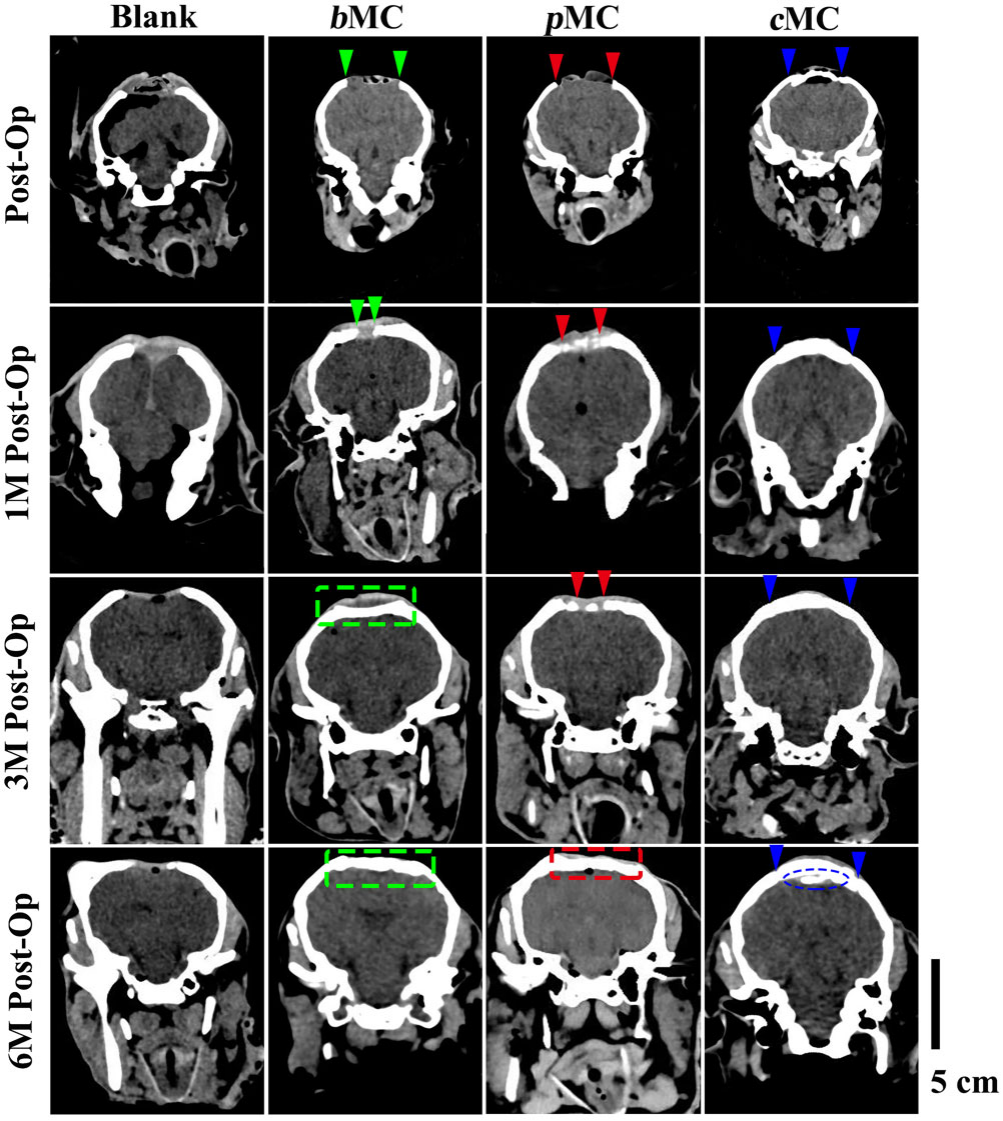
Coronal CT images of the defect area in different groups (blank, *b*MC, *p*MC and *c*MC) postoperatively and at 1 month (1M Post-Op), 3 months (3M Post-Op) and 6 months (6M Post-Op) Scale bar =5 cm. The green, red and blue arrows represent the defect edge positions of *b*MC, *p*MC and *c*MC groups, respectively, and the green boxes, red box and blue circle represent the nascent bone in the defect area in *b*MC, *p*MC and *c*MC groups, respectively.

The coronal CT images of the defect areas in different groups were presented in Fig. 6. The green, red and blue arrows were used to represent the edge positions of the defects in the *b*MC, *p*MC and *c*MC groups, respectively, and the green, red and blue dashed lines were used to represent the nascent bone defects in different groups. At 1 month after the operation, the overall thickness of the cranium in each experimental group was obviously increased, and the size of the defect area in the blank group was basically unchanged. In the *b*MC group, a more obvious increase in density can be observed, and the bone bridges were connected together and span a longer distance. The density increase in the *p*MC group was slighter than that in the *b*MC group, but high-density plaques could be observed to distribute in the repair area. The *c*MC group showed improved fusion with the peripheral cranial bone, with two regenerated parts forming a certain arc. As shown in the green dashed box in Fig. 6, the density of the repair area in the *b*MC group at 3 months after surgery had achieved an overall increase, and the defect area was filled with nascent bone tissue. In addition, the scaffold had not been completely degraded, and a certain scaffold profile was maintained, which was consistent with the results observed in 3D CT. At 3 months after surgery, *p*MC group showed a similar bone bridge connection to the *b*MC group at 1 month postoperatively, which filled in most of the defect area. The scaffold in the *c*MC group had achieved complete bone fusion with the peripheral bone, indicated by the disappearance of the boundary between the scaffold and the cranial bone. After 6 months, a small amount of osteogenic plaque appeared at the edge of the defect in the blank group. The repair area of the *b*MC group was further thickened, exhibiting a tendency to form a curvature similar to the cranial bone. The *p*MC group also achieved a comprehensive increase on bone density in the entire area. The bone bridge was connected into a complete layer, but the shape matching with the surrounding bone was worse than the *b*MC group. The formation of nascent high-density tissue can be observed beneath the *c*MC scaffold.

The *p*MC scaffolds have more efficient osteogenic efficiency. Because of the dense structure of the *c*MC scaffold, the bone repair in the defect area of the *c*MC group can only be performed through a single dura-derived osteogenesis. Different from the dense structure of *c*MC scaffold, *p*MC scaffold is filled with interconnected porous structure, which provides enough space for bone tissue to grow into the material. Compared with a single dura-derived osteogenic pathway, the *p*MC scaffold provides a double-layer osteogenic and dura-derived osteogenic regeneration pathway with a rich nutritional supply. In addition, with the increase of implantation time, *p*MC scaffold were biodegradable and replaced by new bone tissue, indicating that the degradation rate was relatively matched with the induced regeneration rate of new bone, which was conducive to tissue growth. However, the compact structure of *c*MC scaffold and slow degradation rate *in vivo* were not conducive to tissue growth into the internal part of the material. Similarly, the *b*MC scaffold combines the advantages of the two MC scaffolds, and the *p*MC phase has the same bone repair effect as the *p*MC scaffold. Since *c*MC phase can maintain the stability of the entire defect-repair area and the structural stability of the new bone, the advantages of *b*MC scaffolds become more prominent with the increase of implantation.

### Head circumference changes with growth and development

**Figure 7.**
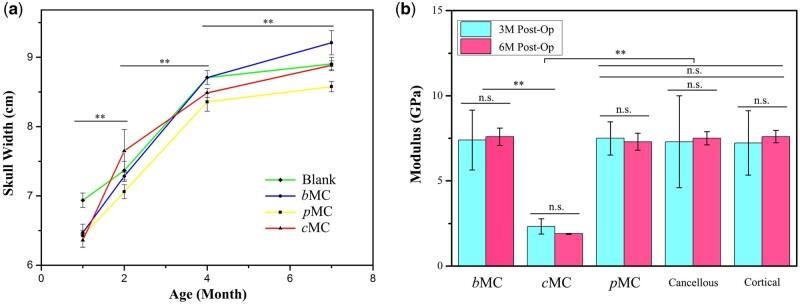
(**a**) Statistical analysis and comparison of the changes of the maximum cranial bone width in blank, *b*MC, *p*MC and *c*MC groups **P <* *0.01, indicating the significant difference between skull widths of the same group at adjacent time points. (**b**) Elastic modulus of nascent bone at 3 and 6 months postoperatively measured by nanoindentation in blank, *b*MC, *p*MC and *c*MC groups. **P <* *0.01, indicating the significant difference between groups at the same time point. n.s. indicating no significant difference in each group at different time points or among the *p*MC, cancellous and cortical groups at the same time point.

The 1-month-old sheep are approximately equivalent to 3–5 years old of human children and are in a period of rapid cranial bone development [31]. The maximum skull widths were measured from the CT images at the different time points, which showed the variation trend of head circumference with the sheep’s development (Fig. 7a). By the end of the experiment, the sheep have grown to 7 months old, and the age has been in the stage of sexual maturity. The whole experiment period ran through the rapid and slow growth and development stages of the sheep. According to the change of head widths, the sheep between 1 and 4 months old were in the rapid growth period, then, the growth rate slowed down after 4 months. The experimental results showed that there was no significant difference on the skull width in different groups at each time point, and the growth and development of the cranial bone was not significantly affected by the implantation of scaffolds.

### Mechanical properties of nascent bone

The nano-mechanical properties of the nascent tissue in the regenerative areas were shown in Fig. 7b. The microscopic elastic modulus of the tissue in defect area of each group was compared with the natural bone at 3 and 6 months after surgery. The modulus of cancellous bone and cortical bone was similar in nano-scale. The results showed that the moduli of nascent tissue in both *b*MC group and *p*MC group were similar with that of the natural bone after 3 or 6 months of repair, which indicated the nascent tissues should be bone tissues with good maturation at 3-month post-operation. In contrast, the elastic modulus in the *c*MC group was much lower than the other groups, indicating the existence of *c*MC that was not degraded and replaced by bone tissues.

### Histological assessment of the regenerated bone

**Figure 8.**
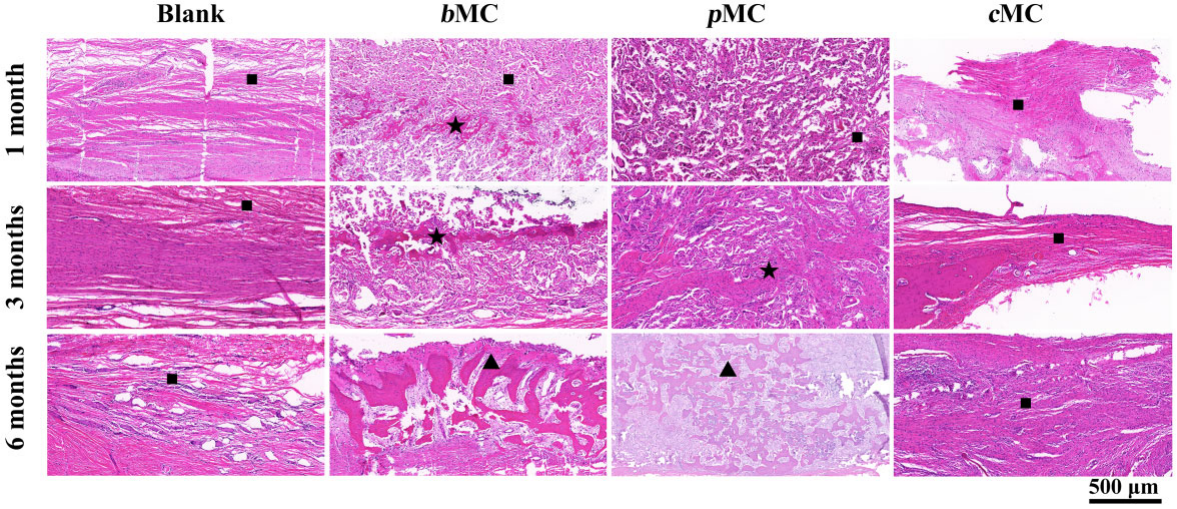
H&E staining of defect area in blank, *b*MC, *p*MC and *c*MC groups at 1, 3 and 6 months postoperatively Scale bar =500 μm. Neo-bone tissues were marked by ★; fibrous tissues were marked by ■; bony trabecular structure was marked by ▲.

At 1, 3 and 6 months, the scaffolds and surrounding tissue in different groups were harvested from the defect regions to evaluate the bone regeneration. Representative cross-sections of the samples stained with H&E were shown in Fig. 8. The pink color of the compact tissue in the slice represented the newly formed bone tissue (marked with ★) that can be easily distinguished from the loose fibrous tissue (marked with ■). At 1 month, consistent with the reconstructed CT images, only a thin layer of fibrous membrane formed in the blank group due to the lack of material bridging, in which the fibers were loose and only a few compact structures. In the *b*MC group, a certain amount of bone tissue can be obviously observed near the dura mater, and the scaffold was well combined with the peripheral primary bone. For the *p*MC group, the outline of the scaffold was still clearly visible, the scaffold material was not completely degraded, and the bone tissue grew into the empty space of the scaffold. There were typical pink staining tissues of nascent bone in the implanted area. In the *c*MC group, due to the high hardness of the material itself, the slices only retained the peripheral tissue of the scaffold and less connective tissue was observed at the interface between the scaffold and the diploic layer, indicating that the scaffold was basically not degraded. However, there was a certain amount of osteogenic structure under the scaffold with fibrous connective tissue as well. After 3 months, the scaffolds in the *c*MC group were still almost not degraded, which were lost during slicing because of the high hardness, and only the surrounding tissues of the scaffolds were retained. There are connective fibrous tissue and nascent bone between the diploic layer and *c*MC scaffold. In the *b*MC group, the area adjacent to the dura mater and the porous part of the scaffold were occupied by osteoid tissue, and obvious trabecular bone structure (marked with circles) could be observed. In addition, most of the scaffold had degraded, especially near the dura mater. In the *p*MC group, nascent bone gradually penetrated into most areas of the defect, and the scaffold was partially degraded into fragments. The adjacent dural area and the center of the scaffold were occupied by bone-like tissue, and the trabecular structure was visible (marked as ▲). In addition, the *p*MC scaffold is almost completely degraded and replaced by regenerated tissue in the region near the dura mater, indicating that the formation of bone tissue mainly occurs through the dura mater pathway. At this time, some of the bones here have matured and formed the same morphology and staining as the original natural bones. In the blank group, the defect area was still dominated by loose fibrous connective tissue, without the formation of bone-like tissue. The fibrous tissue adjacent to the plate barrier was denser than that in the central area of the defect, and had no obvious boundary with the edge of the defect. At 6 months after operation, most of the scaffold materials in the *b*MC group were degraded, and the defect area was filled with new trabecular bone structures, especially in the area between the scaffold and the dura mater, which had been completely occupied by nascent bone tissues. The diploic layer at the edge of the defect was closely connected with *b*MC scaffold, and grew into the scaffold. The color and structure were consistent with the surrounding normal tissues. Similarly, most of the materials in the *p*MC group after 6 months of implantation have been degraded, and the regeneration of nascent bone tissue growing into the *p*MC group is slightly worse than that in the *b*MC group. The number of bone trabecular structures visible in the defect area was less than that in the *b*MC group, and it was relatively loose. Bone trabecular structure and tight fibrous tissue were observed between *p*MC scaffold and dura mater, and the layer was closely connected with the barrier layer. More trabecular bone and nascent bones that grow into the scaffold were observed near the barrier layer, indicating that *p*MC scaffolds promote bone regeneration primarily through diploic layer osteogenesis. The *c*MC scaffold is almost intact and has no obvious gap with surrounding tissues. The fibrous tissue near the barrier layer and below the scaffold was tighter than that in previous time points, with a small amount of mineralized structure formed.

**Figure 9.**
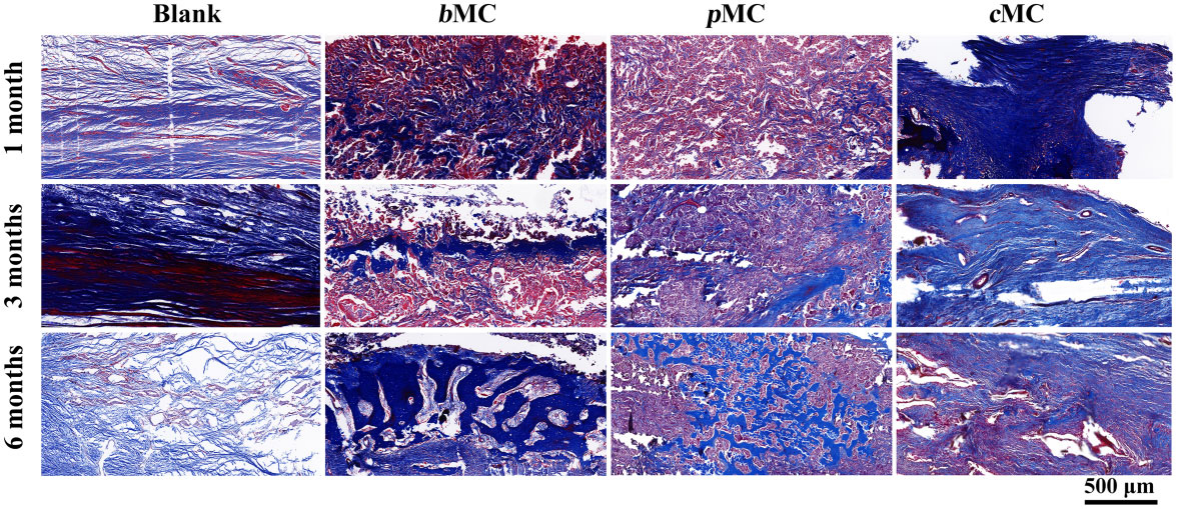
Masson’s trichrome staining of defect area in blank, *b*MC, *p*MC and *c*MC groups at 1, 3 and 6 months postoperatively Scale bar =500 μm.

Masson’s trichrome staining was used to evaluate the maturity level of regenerated tissue in the defect area (Fig. 9). In the representative images, immature nascent bone was in blue, and mature nascent bone was in dark red. From 1 to 6 months after surgery, the white area representing the implanted scaffold in the *b*MC and *p*MC groups became smaller, while the regenerated tissue area became larger, and the change in the *b*MC group was particularly obvious. Loose fibrous connective tissue was gradually replaced by immature trabecular bone and mature bone. Since the scaffold in the *c*MC group had almost no degradation within 6 months after implantation and did not provide sufficient space for the regeneration of nascent bone, neither nascent bone nor mature bone were observed in the defect area in 6 months, and there was no significant change in the surrounding tissues, and fibrous connective tissue remained the dominant position. There was no significant change in the defect area in the blank group, and the loose fibrous connective tissue on the dural side gradually thickened and became dense. After 6 months, the dura mater side of the implant showed the same staining pattern and color as the original bone of the *b*MC scaffold, and the scaffold in the defect area also showed a large number of tissues with the same staining as the surrounding bone. The mature nascent bone was connected above the dura mater and formed a repaired bone layer, showing the highest regeneration level in all groups.

## Discussion

In general, tissue-engineered bone scaffolds with appropriate mechanical properties, microstructure and suitable biodegradation rate that matches the replacement rate of nascent bone is crucial to induce the formation of nascent bone and thus achieve ideal regeneration outcomes. Porous structure can provide large specific surface area and is conducive to the 3D growth of tissue and the diffusion of nutrients and wastes, which is commonly required for scaffolds for bone regeneration [32]. The porosity of tissue-engineered scaffolds is often closely related to the effect of bone repair. In most bone regeneration studies, rapidly biodegradable porous scaffolds have been widely used, which inevitably cannot provide enough mechanical support, e.g. injectable hydrogel [33], pure bioceramic [34], etc. In the case of repairing large cranial bone defects, the defect area is usually about dozens of square centimeters or even more than 100 cm^2^, and the scaffolds have to be fabricated to the sheet structure to adapt to the defects, which puts forward higher requirements for the biomechanical characteristics of the scaffold throughout the repair process. In addition, with the increase of porosity, the strength of the scaffold decreased significantly so that it could not meet the mechanical requirements for cranioplasty. In our previous work, we developed *p*MC scaffolds with suitable porosity and *c*MC scaffolds with dense structure and high strength. Although *p*MC had better osteogenic effect than *c*MC, the typical pure porous scaffold definitely could not meet the mechanical requirements of cranial bone formation to maintain long-term structural stability of nascent bone in entire defect area.

Ideally, the implantable artificial cranium scaffold should be similar to the surrounding natural bone in biomechanics and conducive to the growth of nascent bone [35, 36]. The formation of nascent bone deformities and excessive density of artificial bone structure may lead to poor bone repair [9, 37]. In this study, the *b*MC scaffolds contain both *p*MC and *c*MC phases that mimic the structure of natural bone cancellous bone and dense bone tissue, respectively. Appropriate porosity of *p*MC phase provides a spatial basis for nascent bone ingrowth, material exchange and cell migration, which has a suitable *in vivo* degradation rate to match the rate of nascent bone regeneration. The introduction of *c*MC phase improves the poor mechanical properties of *p*MC phase. According to previous studies, the mechanical strength of the *c*MC structure is close to that of natural bone tissue, which can maintain the structural stability of the scaffold and facilitate the structural stability and integrity of the regenerated nascent bone. Therefore, the *b*MC scaffold has more appropriate biomechanical properties and performs better in osteogenic induction and bone integration.

MC showed good bioactivity and osteogenic activity in promoting cell growth and osteogenesis *in vitro* and *in vivo*. The osteogenic gene expression of BMSCs on PCL, MC/PCL and HA/PCL scaffolds revealed that MC had higher osteogenic activity than HA. BMP-2 was considered as an early marker of bone formation and a signal molecule to promote ALP expression [38]. The BMP-2 gene and its downstream key gene Runx2 were significantly up-regulated on MC/PCL, indicating that MC could induce osteogenic differentiation of BMSCs through BMP signaling pathway. Similarly, MC also promoted the up-regulation of ALP and Col I genes. Col I is a late marker of bone formation, a mineralization template of inorganic phase-calcium phosphate mineral in the process of biomineralization, which provides calcium ion adsorption sites and regulates crystal structure [39, 40]. ALP, which is synthesized and secreted from the precursor state of bone cells to the mature state, is an early marker of osteogenesis, which plays an important role in the process of bone calcification [38, 41, 42]. The up-regulation of ALP could directly explain the osteogenic differentiation of BMSCs. OPN plays an important role in the process of bone formation, containing the acidic domain that interacts with the mineral surface of the extracellular matrix, which can also promote the mineralization of inorganic phase and realize cell ossification [43].

The microenvironment for cranium regeneration requires adequate blood supply, appropriate stem cell sources and appropriate scaffold structure (such as porosity) [44]. Cranium, with special structure, is different from other limb or trunk bones, which is composed of two layers of thick dense bones with a piece of cancellous bone called diploic layer in the middle. Due to the lack of sufficient blood supply and bone marrow, compared with tubular bone, cranial bone has poor self-healing ability and low regeneration rate [45]. Previous results showed that the 30-mm cranial bone defect in sheep beyond critical size could not heal itself. However, different degrees of cranial bone regeneration were observed in the defects of *p*MC, *c*MC and *b*MC groups. Studies have shown that the cranial bone regeneration might involve three pathways: periosteal-induced osteogenesis, diploic layer osteogenesis and dura-derived osteogenesis [25, 26]. In the process of surgical modeling, the periosteum in the defect area was removed, and the growth of nascent bone tissue could only be promoted by diploic layer and/or intact dura mater. Large bone defects are difficult to recover itself because osteoblasts cannot migrate to large defects; therefore, material bridge is required to promote cell migration. In the blank group without scaffold implantation, there was no bridge for osteoblasts to migrate to the defect, leaving only a small amount of vacancies formed by nascent bones at the edge. Only a thin layer of soft tissue was observed in the defect by the naked eye with original cranial bone at both ends, and there was no obvious mineral deposition.

The *p*MC group showed higher bone mineral density, bone bridge length and the more mature nascent bone than the *c*MC group at each time point. According to the CT reconstruction and histological analysis, the amount of nascent bone on the dural side was significantly higher than that on the periosteal side, indicating that the dural osteogenesis efficiency of the *p*MC group was significantly better than that of the periosteal osteogenesis. This phenomenon could be attributed to relatively excellent blood supply and periosteum layer of dura mater. Compared with single dura-derived osteogenesis, the two cranial regeneration pathways are significantly more complex, with a rich nutritional supply, but fewer stem cells [25, 26]. These two pathways have their own advantages, but in our study, it was found that in the *p*MC group, the bone was regenerated through diploic layer osteogenesis and dura mater osteogenesis. More importantly, in the long term, the bone tissue formed near the dura mater was more mature than that formed in the porous scaffold, while the bone tissue between the cancellous bone diploic layer and the degradation scaffold was still immature, which suggested that dura induced nascent bone formation more effectively. Bone bridging occurred across the entire defect above the dura mater, and the histology also supported this observation. With the increase of implantation time, some parts of the *p*MC scaffolds were replaced by nascent bone tissue with the biodegradation process *in vivo*, indicating that the degradation rate relatively matched with the rate of inducing nascent bone regeneration and was in a reasonable range [46]. There was no case that bone formation was not timely due to excessive degradation, or bone tissue was difficult to grow due to excessive degradation. However, it is worth noting that the long-term implantation of *p*MC scaffold also exposed the shortcomings of unstable osteogenic efficiency, and the osteogenic speed of *p*MC scaffold was relatively slow. In addition, due to the insufficient strength of *p*MC scaffold in the experiment, there were cases of death caused by the collision of defect areas between sheep.

In the *c*MC group, through dura-derived osteogenic pathway, a nascent layer of bone was rapidly formed below *c*MC, which could provide long-term and stable biomechanical support instead of *c*MC. In addition, the *c*MC scaffold had good osteoconductivity, mainly through dura-derived osteogenic pathway to induce nascent bone formation. At the late stage of implantation, a certain degree of swelling and slight degradation on the scaffold surface could be observed, and the formation of rough material surface was conducive to bone integration. The X-ray coronal scanning images showed that the scaffold materials were closely combined with the surrounding bone tissue. Since the mechanical properties of the *c*MC scaffold were close to those of the natural bone tissue, the relative integrity maintained for a long time after implantation to ensure the structural integrity of nascent bone formation. Histological results also confirmed the binding of *c*MC to surrounding bone tissue. Nonetheless, there was no obvious biodegradation of the scaffold during the whole implantation, so the inward growth of the surrounding nascent bone was limited. Because of the pore-free structure and slow *in vivo* degradation rate of *c*MC scaffolds, it was difficult for bone to be regenerated into scaffolds through diploic layer pathways before degradation.

The *b*MC scaffold combined the advantages of the two MC bone materials. The *p*MC phase provided sufficient space for the inner growth of a large number of nascent bone tissue and promoted the osteoconductive effect of the scaffold. Because the *p*MC phase was completely wrapped in the *c*MC phase, and was directly contacted with the cranial bone defect edge and dura mater, the regenerated bone tissue could quickly migrate to the pores of *b*MC scaffold through the diploic layer and dura mater osteogenic pathway. The early osteogenic effect of *b*MC scaffold was also close to that of *p*MC scaffold. With the increase of implantation time, compared with the pure *p*MC scaffold, *b*MC scaffold achieved more rapid osteogenesis, which basically improved the bone mineral density in the defect area to a degree similar to that of the peripheral bone at 3 months after operation. The micromechanical strength of the nascent bone tissue was consistent with that of the natural dense bone. The reason is that the *c*MC part in the scaffold could provide certain mechanical support to maintain the structural stability of the nascent bone. By measuring the size of the small sheep’s skull, it could be found that the period of rapid growth and development of the cranial bone before sexual maturity (7 months) and 3 months after operation was the most suitable stage for endogenous osteoblasts to migrate into the material area and differentiate into bone cells. Compared with the *p*MC group, the cycle of bone regeneration induced by *b*MC group was more consistent with the growth and development of sheep. Most importantly, in the later stage of repair, the focus of repair shifted from ossification within the material to building the integrity of the overall structure. Compared with *p*MC group, the coronal X-ray and CT reconstruction images of *b*MC group confirmed its advantage.

In the past decades, various biomaterials have been studied for repairing large cranial bone defects, such as bioceramics, alloys, natural biomaterials and hydrogels, which have good biocompatibility [47–49]. However, their respective shortcomings largely limit their clinical application in the treatment of cranial bone defects. For example, in clinical skull repair cases, pure bioceramic porous scaffolds have low biodegradability and strength [50]. In contrast, alloy implants can distribute stress uniformly along the interface due to their high material strength, but they are not conducive to the reconstruction of new bone due to their poor osteoinductivity [51]. Pure hydrogel has poor mechanical features and fast degradation rate, which cannot be used for the repair and treatment of skull defect cases in clinic [52–56]. However, bioceramic/polymer composite scaffolds have attracted more and more attention due to their excellent bone induction and more suitable mechanical properties. Our work provided an idea for the construction of skull repair scaffolds. MC-based composites were used to construct biphasic composite scaffolds mimicking the characteristics of cancellous bone and dense bone. It was proved that *b*MC was an organic combination of two bionic bone structures. The appropriate *in vivo* degradation rate and interconnected porous structure of *p*MC phase ensured the growth of nascent bone, and *c*MC phase scaffold could provide necessary biomechanical support for cranial bone repair. From the long-term repair effect, *b*MC scaffold was conducive to maintaining the long-term frame stability of defect-repair area. However, some shortcomings of this composite structural material could still be seen. For example, in the case shown in CT reconstruction results in Supplementary Fig. S3, although the overall bone regeneration was good after 6 months of repair, the repair effect in the central region was not good due to the blocking effect of the dense part. Therefore, the structural design of the biphasic composite needs more optimizations according to different *in vivo* cases.

## Conclusion

In this study, a biodegradable *b*MC composite bone scaffold with both cortical bone-like and cancellous bone-like structural features was constructed for repairing large-sized cranial bone defects in developing sheep. The *p*MC phase has interconnected porous structure, similar to the natural cancellous bone structure, which is beneficial to promote host cell homing and metabolic potential. The *c*MC phase, with dense and pore-free structure, has the comparable mechanical properties and density as natural cortical bone. In the developing sheep cranial bone defect model, the bone regeneration effect of *b*MC scaffold was significantly better than that of other groups at the same time point. The *b*MC and *p*MC scaffolds with porous structure promoted bone regeneration through dura-derived and diploic layer pathway, while the *c*MC scaffold was almost non-degradable, leaving no space for new tissue to grow inward, which mainly promoted bone formation under the scaffold through dura. Besides, the *b*MC scaffold possessed improved mechanical properties than *p*MC scaffold and ensured the long-term structural stability of the defect area after implantation, and showed no negative effect on the normal growth and development of the cranial bone.

## Supplementary data

Supplementary data are available at *REGBIO* online.

*Conflict of interest statement*. None declared.

## Funding

We acknowledge the financial support from the National Key R&D Program of China (2020YFC1107602), Shandong Province Key R&D Program of China (2019JZZY011106), and the National Natural Science Foundation of China (No. 81660214, 81960238, 82160250).

## Supplementary Material

rbac004_Supplementary_Data
