# Supplementary material for: Biphasic mineralized collagen-based composite scaffold for cranial bone regeneration in developing sheep
Source: Regen Biomater. 2022 Jan 18;9:rbac004. doi: 10.1093/rb/rbac004 (PMC9113234; doi:10.1093/rb/rbac004)
Supplement: rbac004_Supplementary_Data [file rbac004_supplementary_data.docx]

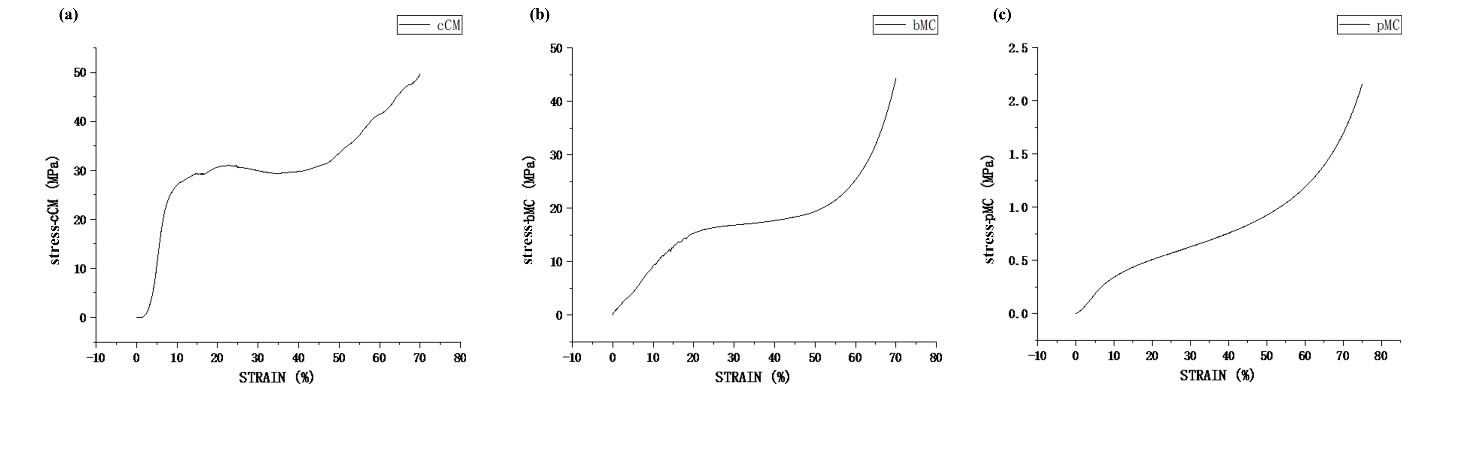


Figure S1. Stress-strain curves of 3 kinds of scaffolds.


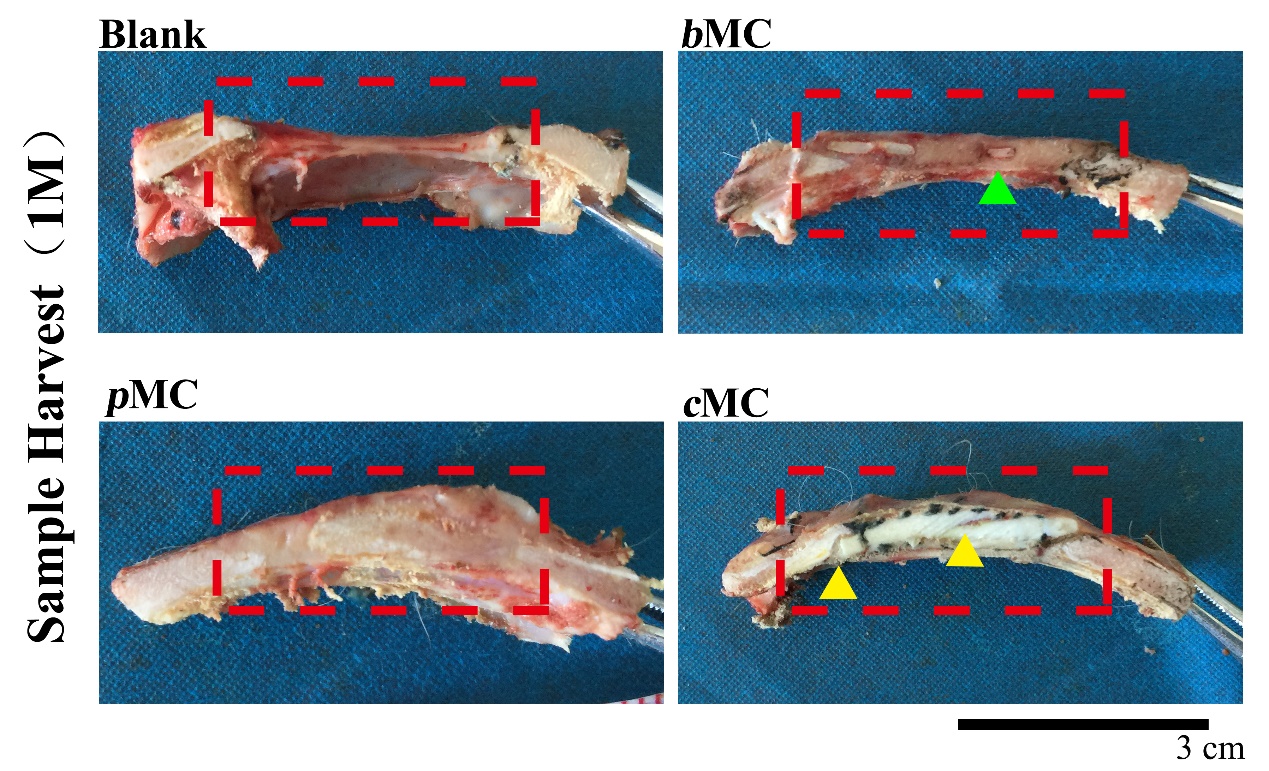


Figure S2. The general morphology of fresh section in defect area of Blank, *b*MC, *p*MC, *c*MC groups.

Table S1.Primer sequences used for RT-PCR gene expression analysis

| Genes | 5’-3’ | Primers |
| --- | --- | --- |
| ALP | Forward Reverse | CCTGGACCTCATCAGCATTT AGGGAAGGGTCAGTCAGGT1T |
| RUNX2 | Forward Reverse | TCTCTGACCGCCTCAGTGATT TGTGTCTGCCTGGGATCTGTA |
| BMP-2 | Forward Reverse | GAAGCCAGGTGTCTCCAAGAG GTGGATGTCCTTTACCGTCGT |
| OPN | Forward Reverse | GGAGTCCGATGAGGCTATCAA TCCGACTGCTCAGTGCTCTC |
| COL-1 | Forward Reverse | TGGATGGCTGCACGAGT TTGGGATGGAGGGAGTTTA |
| OCN | Forward Reverse | GACCCTCTCTCTGCTCACTCT  GACCTTACTGCCCTCCTGCTTG |


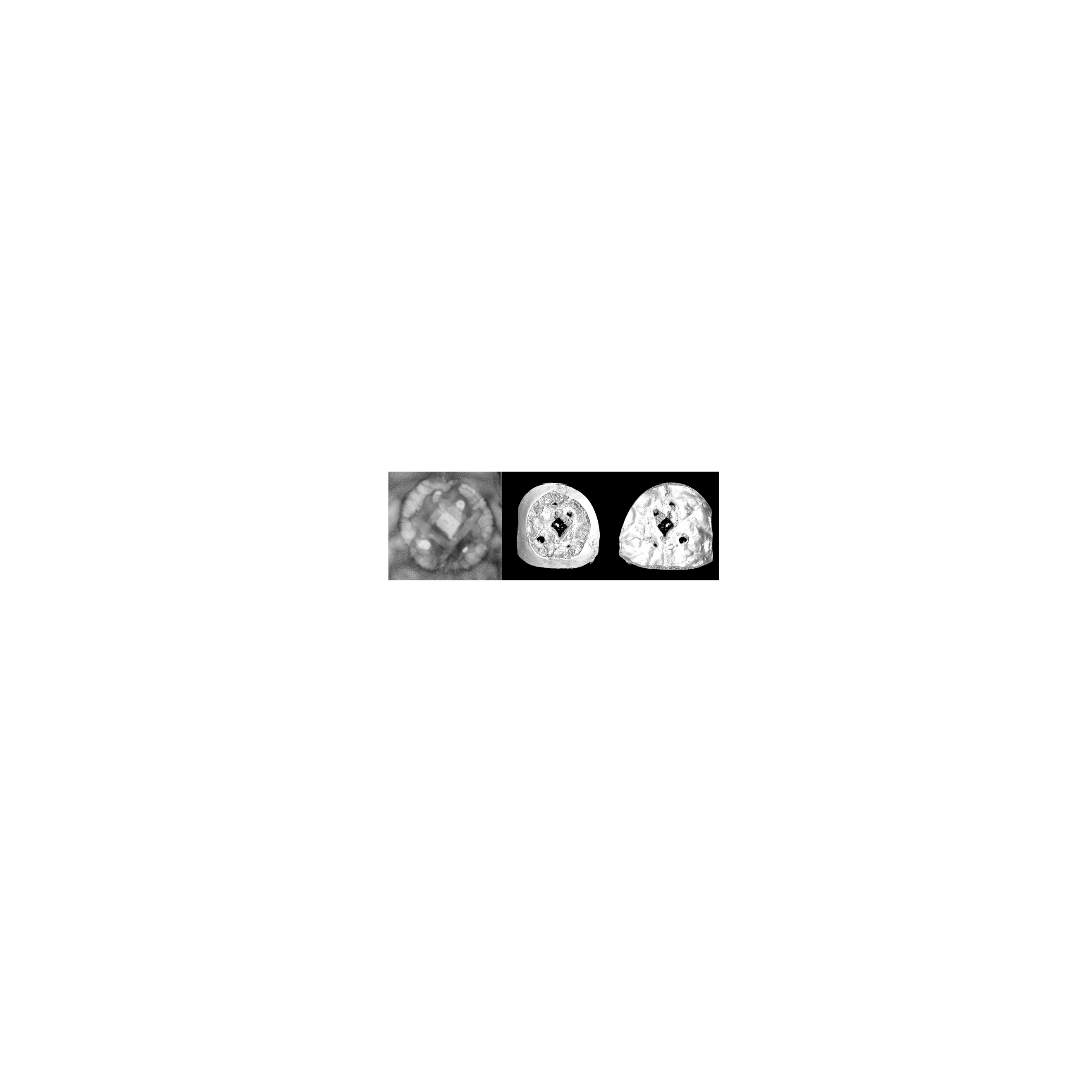


Figure S3. CT reconstruction results of individual *b*MC groups after 6 months of repair
